# The Stoor Hobbit of Guangdong: *Goniurosaurus gollum* sp. nov., a cave-dwelling Leopard Gecko (Squamata, Eublepharidae) from South China

**DOI:** 10.3897/zookeys.991.54935

**Published:** 2020-11-11

**Authors:** Shuo Qi, Jian Wang, L. Lee Grismer, Hong-Hui Chen, Zhi-Tong Lyu, Ying-Yong Wang

**Affiliations:** 1 State Key Laboratory of Biocontrol/ The Museum of Biology, School of Life Sciences, Sun Yat-sen University, Guangzhou, Guangdong 510275, China; 2 Herpetology Laboratory, Department of Biology, La Sierra University, Riverside, California 92515, USA

**Keywords:** *Goniurosaurus gollum*, sp. nov., karst, limestone cave, taxonomy, systematics

## Abstract

A new species of the genus *Goniurosaurus* is described based on three specimens collected from a limestone cave in Huaiji County, Guangdong Province, China. Based on molecular phylogenetic analyses, the new species is nested within the *Goniurosaurus yingdeensis* species group. However, morphological analyses cannot ascribe it to any known species of that group. It is distinguished from the other three species in the group by a combination of the following characters: scales around midbody 121–128; dorsal tubercle rows at midbody 16–17; presence of 10–11 precloacal pores in males, and absent in females; nuchal loop and body bands immaculate, without black spots; iris orange, gradually darker on both sides. The discovery of yet another limestone-adapted species of *Goniurosaurus* in Guangdong Province underscores a growing body of evidence for the high biodiversity of limestone habitats and brings into sharp focus the urgent need for their conservation.

## Introduction

The eublepharid genus *Goniurosaurus* Barbour, 1908, currently contains 22 species that are scattered throughout much East and northern Southeast Asia (Uetz et al. 2020; Zhu et al. 2020; Qi et al. 2020). All species of *Goniurosaurus* have generally restricted, circumscribed ranges and many are restricted to habitats with either granite or limestone (karst) rock (Wang et al. 2014; Liang et al. 2018; Qi et al. 2020). Recent molecular phylogenetic analyses resulted in the partitioning of *Goniurosaurus* into four monophyletic species groups (Liang et al. 2018 ; Qi et al. 2020; Zhu et al. 2020), namely the *G. kuroiwae*, *G. lichtenfelderi*, *G. luii* and, *G. yingdeensis* groups. The morphological comparisons also sustain the recognition of the *G. kuroiwae* and *G. yingdeensis* groups, however, the definitions of the *G. lichtenfelderi* and *G. luii* groups still require clarification (Qi et al. 2020).

Currently, three narrowly distributed species within the *Goniurosaurus yingdeensis* group are known from the karst environments of northern Guangdong, China: *G. yingdeensis* Wang, Yang & Cui, 2010, *G. zhelongi* Wang, Jin, Li & Grismer, 2014 and *G. varius* Qi, Grismer, Lyu, Zhang, Li & Wang, 2020. These three species share consistent morphological features that differentiate them from species of the other groups: (1) base of claws sheathed by four scales, two lateral scales of claw short and shell-shaped; (2) precloacal pores fewer than 15 in males and absent in most females (precloacal pores present in females in *G. yingdeensis* only); precloacal pores form a continuous transverse series not extending onto the femora; (3) enlarged row of supraorbital tubercles indistinct or absent; (4) nuchal loop rounded posteriorly; and (5) four body bands between the nuchal loop and the caudal constriction.

During our herpetological surveys in Guangdong Province, China, three specimens of *Goniurosaurus* were collected from a new locality during May 2020. Morphological and molecular analyses place this new population within the *Goniurosaurus yingdeensis* group but cannot ascribe them to any of the known species. We therefore describe this population as a new species.

## Materials and methods

### Sampling

Three specimens of the undescribed species were collected from Huaiji County, Guangdong Province, China. Following euthanasia, all specimens were fixed in 10% formalin and transferred to 75% alcohol. Tissue samples were preserved in 99% alcohol and stored at -40 °C. All specimens are deposited in The Museum of Biology, Sun Yat-sen University (**SYS**). Sequences of other species of *Goniurosaurus* generated by Qi et al. (2020) were accessed from GenBank.

### Morphological characters

Measurements were taken following Ziegler et al. (2008) using digital calipers (Neiko 01407A Stainless Steel 6-Inch Digital Caliper, USA) to the nearest 0.1 mm. Abbreviations of morphological characters are as follows: **SVL** snout-vent length (from tip of snout to vent); **TaL** tail length (from vent to tip of tail); **HL** head length (from tip of snout to posterior margin of ear opening); **HW** maximum head width; **SE** snout-to-eye distance (measured from tip of snout to the boney anterior margin of the orbit); **EE** eye-to-ear distance (from the boney posterior margin of the orbit to posterior margin of ear opening); **SPL** supralabials; **IFL** infralabials; **N** nasal scales surrounding nare; **IN** internasals; **PostIN** granular scales bordering the internasals; **PM** postmentals; **GP** gular scales bordering postmentals; **CIL** eyelid fringe scales or ciliaria; **PO** preorbital scales (number of scales in a line from posterior margin of external naris to anterior margin of the boney orbit); **GST** granular scales surrounding dorsal tubercles; **PTL** paravertebral tubercles between limb insertions; **DTR** longitudinal dorsal tubercle rows at midbody; **MB** scales around midbody; **PP** precloacal pores; **PAT** postcloacal tubercles. Bilateral scale counts are given as left/right.

Morphological character data of known congeners were taken from the literature (Grismer et al. 1999, 2002; Orlov et al. 2008; Ziegler et al. 2008; Blair et al. 2009; Wang et al. 2010, 2013, 2014; Yang and Chan 2015; Zhou et al. 2018; Qi et al. 2020; Zhu et al. 2020) and from 34 examined museum specimens of the eight species listed in the Appendix.

### DNA Extraction, Polymerase Chain Reaction (PCR), and sequencing

Genomic DNA was extracted from muscle tissue samples, using a DNA extraction kit from Tiangen Biotech (Beijing) Co., Ltd. Partial segments of the mitochondrial genes 16S ribosomal RNA gene (16S) and Cytochrome b gene (Cytb) were amplified. Primers used for 16S were r16S-5L (5’- GGTMMYGCCTGCCCAGTG -3’) and 16sbr-H (5’- CCGGTCTGAACTCAGATCACGT-3’) (Palumbi et al. 1991) and for Cytb the primers were L14731 (5’- TGGTCTGAAAAACCATTGTTG-3’) (Honda et al. 2014) and H15149m (5’- GCMCCTCAGAAKGATATTTGYCCTCA-3’) (Chambers and MacAvoy 1999). The PCR procedure was performed with an initial denaturation at 94 °C for 5 min, 35 cycles of 94 °C for 30 s, 55 °C for 30 s and 72 °C for 1 min, followed by a final extension at 72 °C for 10 min (Liang et al. 2018). PCR products were purified with spin columns and then sequenced with forward primers using BigDye Terminator Cycle Sequencing Kit as per the guidelines on an ABI Prism 3730 automated DNA sequencer by Shanghai Majorbio Bio-pharm Technology Co., Ltd.

### Phylogenetic analysis

Fifty-one sequences from 13 known *Goniurosaurus* species plus one outgroup sequence from the eublepharid *Hemitheconyx taylori* Parker, 1930, which was used to root the tree, were obtained from GenBank and composed the dataset (Table 1). DNA sequences were aligned by the Clustal W with default parameters (Thompson et al. 1997) and trimmed with gaps partially deleted in MEGA 6 (Tamura et al. 2013). Two gene segments, with 486 base pairs (bp) of 16S and 396 bp of Cytb, were concatenated seriatim into an 882 bp sequence, and further divided into two partitions based upon each gene. The partitions were tested in jmodeltest v2.1.2 with Akaike and Bayesian information criteria, all resulting the best-fitting nucleotide substitution models of GTR+I+G. Sequence data were analyzed using Bayesian inference (BI) in MrBayes 3.2.4 (Ronquist et al. 2012), and maximum likelihood (ML) in RaxmlGUI 1.3 (Silvestro and Michalak 2012). Two independent runs were conducted in the BI analysis with 10,000,000 generations each and sampled every 1000 generations with the first 25% of samples discarded as burn-in, resulting in a potential scale reduction factor (PSRF) of < 0.005. In the ML analysis, a bootstrap consensus tree inferred from 1000 replicates was generated. Nodes with Bayesian posterior probabilities (BPP) ≥0.95 and ML support values of ≥70 were considered strongly supported (Huelsenbeck et al. 2001; Wilcox et al. 2002). Uncorrected pairwise sequence divergences utilizing the 16s gene were calculated using MEGA 6.

**Table 1.** Localities, voucher information, and GenBank accession numbers for all specimens used in this study.

Species name	Locality	Specimen voucher	16S	Cytb	References
**Ingroup: *Goniurosaurus***					
***Goniurosaurus yingdeensis* species group**					
(1) *Goniurosaurus gollum* sp. nov.	Huaiji, Guangdong, China	SYS r002420	MT995784	MT995787	This study
(2) *Goniurosaurus gollum* sp. nov.	Huaiji, Guangdong, China	SYS r002421	MT995785	MT995788	This study
(3) *Goniurosaurus gollum* sp. nov.	Huaiji, Guangdong, China	SYS r002422	MT995786	MT995789	This study
(4) *G. varius*	Yangshan, Guangdong, China	SYS r002330	MT995753	MT995768	Qi et al. 2020
(5) *G. varius*	Yangshan, Guangdong, China	SYS r002331	MT995754	MT995769	Qi et al. 2020
(6) *G. varius*	Yangshan, Guangdong, China	SYS r002333	MT995755	MT995770	Qi et al. 2020
(7) *G. varius*	Yangshan, Guangdong, China	SYS r002362	MT995756	MT995771	Qi et al. 2020
(8) *G. varius*	Yangshan, Guangdong, China	SYS r002363	MT995757	MT995772	Qi et al. 2020
(9) *G. yingdeensis*	Yingde, Guangdong, China	SYS r001271	MT995759	MT995774	Qi et al. 2020
(10) *G. yingdeensis*	Yingde, Guangdong, China	SYS r001272	MT995760	MT995775	Qi et al. 2020
(11) *G. yingdeensis*	Yingde, Guangdong, China	SYS r001493	MT995761	MT995776	Qi et al. 2020
(12) *G. yingdeensis*	Yingde, Guangdong, China	SYS r002115	MT995762	MT995777	Qi et al. 2020
(13) *G. zhelongi*	Yingde, Guangdong, China	SYS r000816	KJ423105	MT995778	Wang et al. 2014; Qi et al. 2020
(14) *G. zhelongi*	Yingde, Guangdong, China	SYS r001491	MT995763	MT995779	Qi et al. 2020
(15) *G. zhelongi*	Yingde, Guangdong, China	SYS r001492	MT995764	MT995780	Qi et al. 2020
(16) *G. zhelongi*	Yingde, Guangdong, China	SYS r002108	MT995765	MT995781	Qi et al. 2020
***Goniurosaurus luii* species group**					
(17) *G. huuliensis*	Vietnam	N/A	AB853453	AB853479	Honda et al. 2014
(18) *G. liboensis*	Libo, Guizhou, China	SYS r000217	KC900230	N/A	Wang et al. 2013
(19) *G. luii*	Jingxi, Guangxi, China	SYS r000255	KC765083	N/A	Wang et al. 2013
(20) *G. luii*	Jingxi, Guangxi, China	SYS r000256	KC765084	N/A	Wang et al. 2013
(21) *G. luii*	Cao Bang,Vietnam	ZFMK 87057	EU499391	N/A	Ziegler et al. 2008
***Goniurosaurus lichtenfelderi* species group**					
(22) *G. bawanglingensis*	Bawangling, Hainan, China	SYS r002162	MT995758	MT995773	Qi et al. 2020
(23) *G. bawanglingensis*	Bawangling, Hainan, China	BL-RBZ-021	MH247190	MH247201	Liang et al. 2018
(24) *G. hainanensis*	Jianfengling, Hainan, China	SYS r000349	KC765080	N/A	Wang et al. 2013
(25) *G. zhoui*	Central area, Hainan, China	SYS r002213	MT995766	MT995782	Qi et al. 2020
(26) *G. zhoui*	Central area, Hainan, China	SYS r002214	MT995767	MT995783	Qi et al. 2020
(27) *G. zhoui*	Central area, Hainan, China	BL-RBZ-001	MH247196	MH247207	Liang et al. 2018
***Goniurosaurus kuroiwae* species group**					
(28) *G. kuroiwae*	Northern Okinawajima Island, Japan	N/A	AB853448	AB853473	Honda et al. 2014
(29) *G. orientalis*	Iejima Island, Japan	N/A	AB853446	AB853467	Honda et al. 2014
(30) *G. splendens*	Tokunoshima Island, Japan	N/A	AB853451	AB853477	Honda et al. 2014
(31) *G. yamashinae*	Kumejima Island, Japan	N/A	AB853442	AB853460	Honda et al. 2014
**Outgroup**					
(32) *Hemitheconyx taylori*	East Africa	N/A	AB308457	N/A	Jonniaux and Kumazawa 2008

## Results

**Figure 1.**
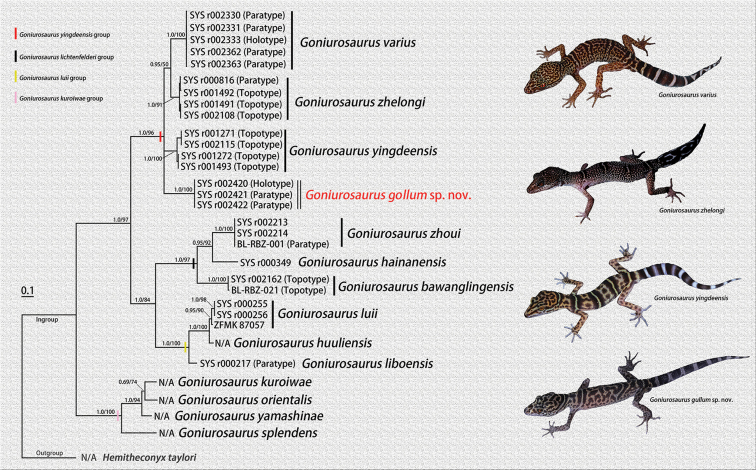
Bayesian inference tree of 14 species of *Goniurosaurus*, based on the partial DNA sequences of the mitochondrial 16S rRNA and Cytb genes. *Hemitheconyx taylori* is the outgroup. Numbers before slash indicate Bayesian posterior probabilities (BPP) and numbers after slash are bootstrap support for ML (1000 replicates) analyses.

The ML and BI analyses resulted in identical topologies (Fig. 1) and maintained a high degree of consistency with recent molecular phylogenetic analyses (Liang et al. 2018; Qi et al. 2020). Uncorrected pairwise sequence divergences are reported in Table 2. The phylogenetic analyses support previous analyses indicating that *Goniurosaurus* can be divided into four strongly supported clades, namely the *G. kuroiwae* group, *G. lichtenfelderi* group, *G. luii* group and the *G. yingdeensis* group. The *Goniurosaurus yingdeensis* group is composed of four species with genetic differences among them ranging from 5.5–5.9%. Three of the species are *G. varius*, *G. yingdeensis* and *G. zhelongi* and the fourth species is composed of the individuals of the new population from Guangdong Province. All specimens were recovered as monophyletic with a high nodal support (1.00 in BI and 100 in ML) and low intrapopulational genetic differentiation (0.0–0.5%; Table 2). But due to low nodal support, the phylogenetic relationships among *G. varius*, *G. yingdeensis* and *G. zhelongi* are still unclear. Additionally, the Guangdong population has a combination of morphological characteristics distinguishing it from other species in the *G. yingdeensis* group as well as showing significant morphological differences from all known congeners. Thus, we hereby describe the specimens from Guangdong Province as a new species.

**Table 2.** Uncorrected *P*-distance of 16S gene among 14 *Goniurosaurus* species used in this study.

ID		1–3	4–8	9–12	13–16	17	18	19–21	22–23	24	25–27	28	29	30	31
**1–3**	*Goniurosaurus gollum* sp. nov.	0–0													
**4–8**	*G. varius*	5.6	0–0.1												
**9–12**	*G. yingdeensis*	5.9	4.2	0–0.3											
**13–16**	*G. zhelongi*	5.5	3.3	4.7	0–0.2										
**17**	*G. huuliensis*	17.4	12.9	15.5	14.5	/									
**18**	*G. liboensis*	15.7	13.1	13.4	13.5	6.1	/								
**19–21**	*G. luii*	16.4	13.9	14.5	14.7	1.4	5.9	0–0.5							
**22–23**	*G. bawanglingensis*	17.3	15.9	16.8	15.8	17.4	14.5	17.3	0–0.3						
**24**	*G. hainanensis*	17.3	16.6	16.6	17.9	14.8	14.5	15.3	8.3	/					
**25–27**	*G. zhoui*	16.7	16.3	18.1	17.3	16.1	16.5	17.2	7.0	7.5	0-0				
**28**	*G. kuroiwae*	22.7	21.2	20.3	22.4	22.2	23.4	21.5	18.7	18.9	18.9	/			
**29**	*G. orientalis*	21.3	18.5	18.2	19.6	21.3	22.2	21.3	19.6	19.8	18.8	3.6	/		
**30**	*G. splendens*	21.9	19.7	19.0	21.8	22.9	23.3	22.2	19.8	18.1	20.2	7	7.9	/	
**31**	*G. yamashinae*	22	18.5	18.8	19.9	21.4	22.9	22	20.3	19.1	19.2	3.7	3.1	7	/

### Goniurosaurus gollum

Taxon classificationAnimaliaSquamataEublepharidae

Qi, Wang, Grismer, Lyu & Wang sp. nov.

8D07A66D-0005-58B5-88FE-D579BEB686B1

http://zoobank.org/C1369EA0-37AB-457C-89C8-E98ED643E1E4

Figs 2 , 3 , 4A , 5

#### Material examined.

**Figure 2.**
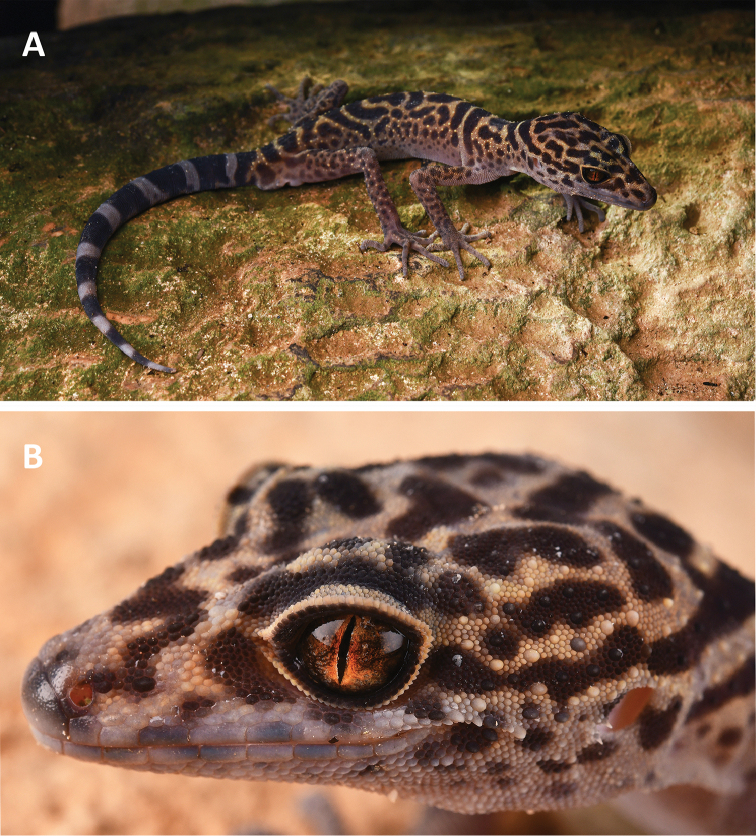
**A** The general aspect of the adult male holotype of *Goniurosaurus gollum* sp. nov. (SYS r002420) in life **B** scalation and coloration characters of the head of the holotype. Photographs by Shuo Qi.

**Figure 3.**
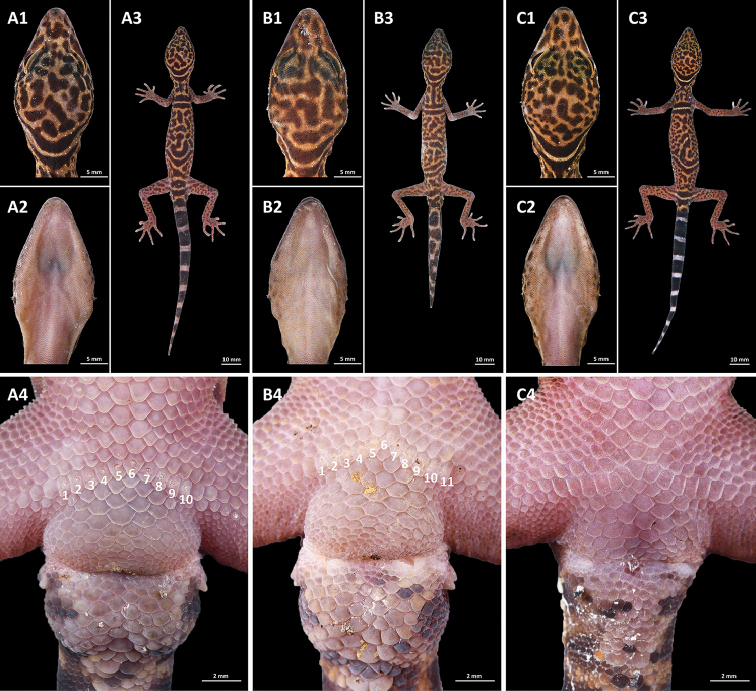
Type series of *Goniurosaurus gollum* sp. nov. **A** holotype, male, SYS r002420 **B** paratype, male, SYS r002421 **C** paratype, female, SYS r002421; (1) dorsal view; (2) dorsal view of head; (3) ventral view of head; (4) close-up of the precloacal region, the Arabic number refer to the number of precloacal pores. Photographs by Shuo Qi.

**Figure 4.**
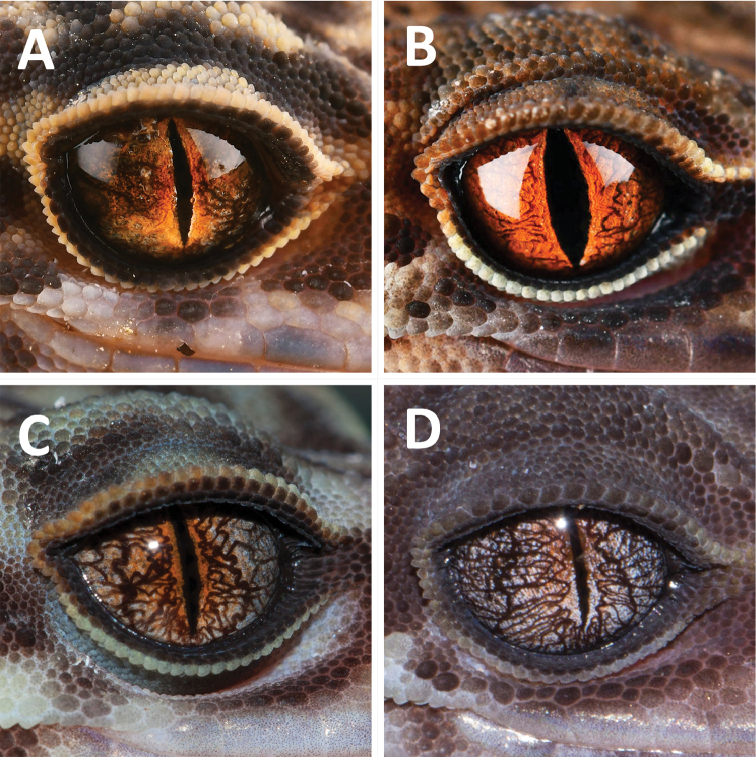
Comparisons of iris color with three closely related congeners **A***Goniurosaurus gollum* sp. nov. (holotype, SYS r002420) **B***Goniurosaurus varius* (holotype, SYS r002333) **C***Goniurosaurus yingdeensis* (holotype SYSr000504) **D***Goniurosaurus zhelongi* (holotype, SYS r000770). Photographs by Shuo Qi and Ying-Yong Wang.

**Figure 5.**
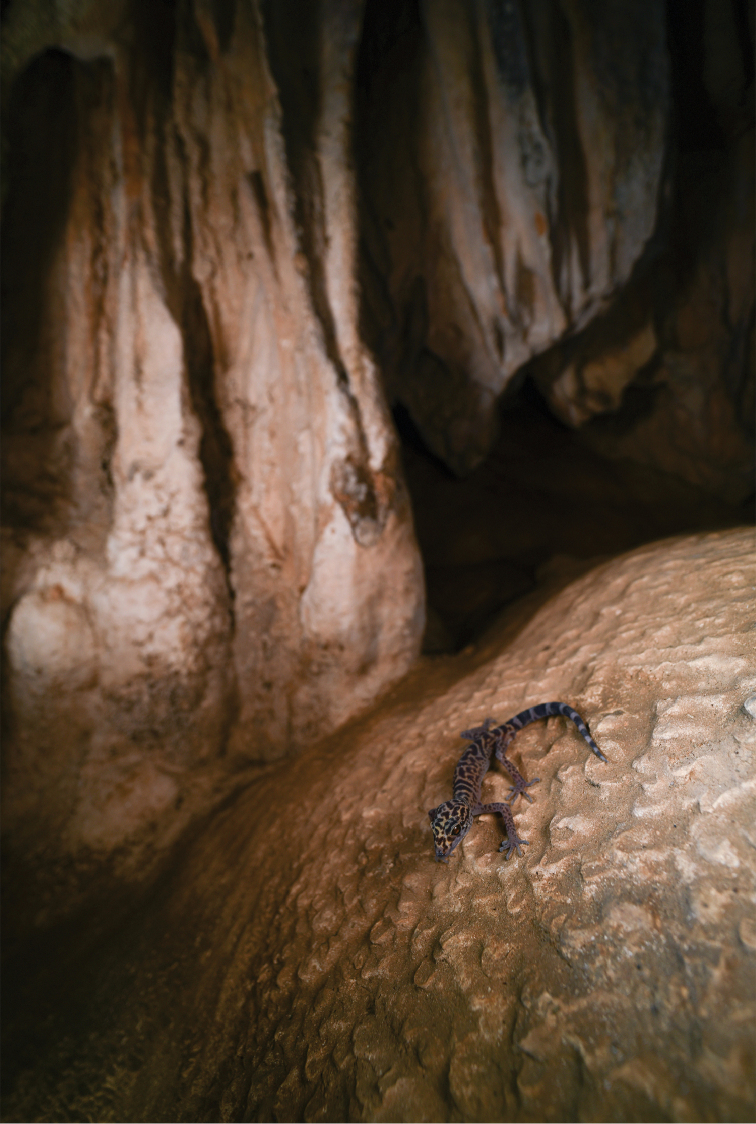
The holotype of *Goniurosaurus gollum* sp. nov. (SYS r002420) at its habitat: a barren limestone cave of Guangdong, China. Photograph by Shuo Qi.

***Holotype*.**SYS r002420, adult male (Figs 2, 3A, 4A, 5), collected by Shuo Qi, Jian Wang and Hong-Hui Chen on 21 May 2020 from Huaiji County, Zhaoqing City, Guangdong Province, China. Exact locality available to only qualified researchers upon request. ***Paratypes*.** One adult male (SYS r002421) and one adult female (SYS r002422) share the same collection information as the holotype.

#### Diagnosis.

*Goniurosaurus gollum* sp. nov. can be distinguished from all other congeners by the following combination of characters: (1) adult body size moderate, 91.0–93.4 mm SVL; (2) nasal scales surrounding nares seven or eight; (4) internasal single; (5) eyelid fringe scales 59–63; (6) granular scales of upper eyelids similar in size to those on top of head; (7) scales around midbody 121–128; (8) longitudinal dorsal tubercle rows at midbody 16 or 17; (9) paravertebral tubercles between limb insertions 25 or 26; (10) claws sheathed by four scales, dorsal claw scales small, two lateral claw scales short and shell-shaped; (11) axillary pockets deep; (12) presence of 10 or 11 precloacal pores in males and absent in females; (13) dorsal ground color of head, body, and limbs in adults yellowish brown and mottled with irregularly shaped dark-brown blotches; (14) nuchal loop complete, rounded posteriorly; (15) presence of three or four thin dorsal body bands between nuchal loop and caudal constriction, with black anterior and posterior borders, bands usually irregularly shaped; (16) iris orange, gradually darker on both sides.

#### Comparisons.

*Goniurosaurus gollum* sp. nov. can be distinguished from the other 21 known species in the genus by the following combination of characters: base of claws being sheathed by four scales, two lateral claw scales short and shell-shaped (vs. claws sheathed by four scales, two lateral scales of claw long, curved in *G. lichtenfelderi* group and *G. luii* group, and not sheathed in *G. kuroiwae* group); having 10 or 11 precloacal pores in males (vs. 17–46 in *G. lichtenfelderi* group, 16–33 in *G. luii* group and absent in *G. kuroiwae* group); and lacking an enlarged row of supraorbital tubercles (present in *G. lichtenfelderi* group and *G. luii* group).

*Goniurosaurus gollum* sp. nov. can be distinguished from its closest relatives in the *Goniurosaurus yingdeensis* group by the following combination of characters: scales around midbody 121–128 (vs. 101–110 in *G. varius*, 101–116 in *G. yingdeensis*, 99–109 in *G. zhelongi*); longitudinal dorsal tubercle rows at midbody 16 or 17 (vs. 21–24 in *G. varius*, 20–25 in *G. yingdeensis*, 23–28 in *G. zhelongi*); absence of ten precloacal pores in females (vs. present in *G. yingdeensis*); nuchal loop and body bands immaculate (vs. having black spots in *G. varius*); iris orange, gradually darker on both sides (vs. iris orange-red in *G. varius*, iris gray and becoming orange near the pupil in *G. yingdeensis*, iris gray-white and tinged with orange in *G. zhelongi*). Additional comparisons of morphological characteristics are provided in Table 4 and Figure 4.

#### Description of holotype.

Adult male with original tail; SVL 93.4 mm; HL 24.2 mm; HW 16.2 mm; SE 9.3 mm; EE 9.3 mm; SVL:HL 3.9; HL:HW 1.5; SE:EE 1. Head triangular, wider than neck, covered with granular scales, densely interspersed with tubercles in the temporal and occipital regions; area between orbits uniformly covered by small granular scales; supraorbital tubercles nearly uniform in size; scales of rostrum slightly larger than those in between orbits; rostral convex and hemi-elliptic, 1.8 times as broad as high, middorsal portion of rostral partially sutured dorsomedially, bordered laterally by first supralabial and prenasal, dorsolaterally by supranasal, dorsally by one internasal; external nares oval, surrounded by 8/9 nasals each, anteriorly by prenasal and supranasal, dorsally by supranasal and a granular scale, posteriorly by 7/8 smaller granular scales, and ventrally by the prenasal; prenasal with long recurved ventral portion; supranasals large, separated by one internasals; supralabials rectangular, 10/10; preorbital scales 16/17; eyes relatively large, pupils vertical; eyelid fringe scales 59/60; outer surface of upper eyelid composed of granular scales of about same size of those on top of head; external auditory meatus circular, tympanum deeply recessed; mental triangular, bordered laterally by first infralabial and posteriorly by three postmentals; infralabials rectangular, 10/10; gular scales juxtaposed uniform granular, abruptly into flat juxtaposed pectoral scales, and grading posteriorly imbricated larger ventral scales. Tongue with a small notch at tip. Crowns of teeth expanded, occlusal margins bearing multiple ridges.

Dorsal surface of neck and body covered with uniform granular scales, interspersed with densely sharply pointed conical tubercles; scales around midbody 125; longitudinal rows of dorsal tubercles at midbody 16; vertebral row of scales indistinct; paravertebral tubercles between limb insertions 25; dorsal body tubercles surrounded by 9–10 granular scales; dorsal scales grading ventrally into larger flattened imbricate ventral scales; ten precloacal pores in a transverse series; postcloacal region greatly swollen, covered with imbricate flattened scales, containing 2/2 postcloacal tubercles laterally at level of the vent.

Tail original, long and thin, thickest at base, bearing whorls anteriorly, gradually narrowing to the tip; composed of nine recognizable annuli anteriorly that are 8–9 scales wide, annuli fade abruptly posteriorly into flat juxtaposed scales; incorporating 2–8 sharply pointed conical tubercles in a transverse row, tubercles do not encircle the tail; ventral caudals larger and more nearly square than dorsal caudals.

Limbs relatively long and slender; dorsal surface covered with granular scales, densely interspersed with tubercles; ventral surface covered by flat scales, juxtaposed, subimbricate or imbricate; dorsal surface of pes and manus covered with granular scales, interspersed with several conical tubercles on top of pes, lacking tubercles on top of manus; hind limbs slightly larger than forelimbs; ventral surfaces of pes and manus covered with large granular scales; axillary pockets deep; subdigital lamellae wide, 10/10 on Finger I, 12/14 on Finger II, 15/16 on Finger III, 16/14 on Finger IV, 13/14 on Finger V, 12/12 on Toe I, 15/ 16 on Toe II, 20 / 19 on Toe III, 22 / 23 on Toe IV, and 18 / 20 on Toe V; fingers laterally compressed, relative finger lengths I<V<II<IV<III; toes laterally compressed, third toe nearly as long as the fourth toe, relative toe length I<II<V<III<IV; base of claws sheathed by four scales, two lateral scales of claw short, asymmetrical shell-shaped.

#### Coloration in life.

Dorsal ground color of head, neck, body, and limbs yellowish brown, mottled with irregularly shaped dark-brown blotches; nuchal loop complete and rounded posteriorly, anterior ends terminating at posterior margins of ear openings, edged dorsally and ventrally by wide dark-brown margin, yellow. Only two complete body bands can be recognized between nuchal loop and caudal constriction: first band located posterior to axilla; second band inserts onto dorsal surface of thigh, bands on limbs dirty yellow, lacking dark spots, edged by broad dark-brown borders anteriorly and posteriorly, other blotches incomplete, not forming a complete bands. Supralabials and infralabials grayish brown; pupils vertical and appear black; iris orange, gradually darkening on both sides; dorsal surface of limbs light grayish brown with dark brown and dirty yellow tubercles and dark spots and blotches; chin, throat, thorax, and ventral surfaces of body pink, tinged brownish, with dark-brown lateral spots; ventral surface of limbs pink, tinged brownish, without dark-brown spots; digits light grayish brown; ground color of original tail dark brown with nine immaculate white caudal bands completely encircling the tail, and a white tip. Body color becomes darker after capture.

#### Coloration in preservative.

Dorsal ground color of head, body, and limbs become darker; ventral surface faded to grayish white; all darker spots, blotches and bands on dorsal surface blurred.

#### Variations.

Measurements of type series specimens are shown in Table 3. Two paratypes have same approximate measurements as holotype, but have significant variations in coloration. Male paratype (SYS r002421, Fig. 3B) has four body bands, the second band extending a three forked branch connecting with third band. Female paratype (SYS r002422, Fig. 3C) has three complete body bands, second band extending a branch forward to middle of body, original tail has ten immaculate white caudal bands completely encircling tail, and dark-brown tip.

**Table 3.** Mensural (mm) and meristic diagnostic characters (minimum/maximum) of type series of *Goniurosaurus gollum* sp. nov. See Materials and methods for abbreviations. * holotype, # paratype.

Morphological character	SYS r002420 *	SYS r002421 #	SYS r002422 #
**Sex**	male	male	female
**SVL**	93.4	93.3	91.0
**TaL**	83.5	Regenerated	76.0
**HL**	24.2	24.0	23.3
**HW**	16.2	15.3	14.8
**SE**	9.3	9.3	9.1
**EE**	9.3	9.4	8.6
**SPL**	10/10	10/10	10/10
**IFL**	10/10	10/10	10/10
**N**	8/9	9/8	8/9
**IN**	1	1	1
**PostIN**	2	2	2
**PM**	2	3	3
**GP**	7	8	8
**CIL**	59/60	59/61	63/63
**PO**	16/17	15/15	16/17
**GST**	9–10	9–10	9–11
**PTL**	25	26	25
**DTR**	16	16	17
**MB**	125	121	128
**PP**	10	11	Absent
**PAT**	2	2	2

#### Etymology.

The specific epithet “*gollum*” is named after the fictional character, Gollum, from J.R.R. Tolkien’s *The Lord of the Rings* book series. This new species and Gollum have similar cave-dwelling habit and emaciated body. We suggest the common name as “Gollum Leopard Gecko”, and according to the type locality, we suggest the Chinese formal name as “guǎng dōng jiǎn hǔ” (广东睑虎).

**Table 4.** Diagnostic characters distinguishing *Goniurosaurus gollum* sp. nov. from all other known species of *Goniurosaurus*. Data come from Grismer et al. 1999, 2002; Orlov et al. 2008; Ziegler et al. 2008; Blair et al. 2009; Wang et al. 2010, 2013, 2014; Yang and Chan 2015; Zhou et al. 2018; Qi et al. 2020; Zhu et al. 2020.

**Character**	***G. kuroiwae* group**	***G. lichtenfelderi* group**	***G. luii* group**	***G. yingdeensis* group (4 species)**			
	**(5 spp.)**	**(5 spp.)**	**(7 spp.)**	***Goniurosaurus gollum* sp. nov.**	***G. varius***	***G. yingdeensis***	***G. zhelongi***
Scales of upper eyelid one-half the size of scales on the top of head or equal in size	Equal	Equal	Equal or 1/2	Equal	Equal	Equal	Equal
Enlarged row of supraorbital tubercles	Absent	Absent or present	Absent or present	Absent	Absent	Absent or indistinct	Absent or indistinct
Eyelid fringe scales	<52	43–77	41–67	59–63	50–56	46–64	42–53
No. of paravertebral tubercles	Unknown	23–36	27–38	25–26	27–29	22–33	28–33
Dorsal tubercle rows at midbody	Unknown	19–22	20–25	16–17	21–24	20–25	23–28
Scales around midbody	Unknown	95–140	112–147	121–128	101–110	101–116	99–109
Nasal scales surrounding nares	Unknown	8–9	5–9	7–8	7–9	7–11	6–8
Internasals	Unknown	1	0–3	1	1–2	1–3	1–2
Tubercles between orbits	Present or absent	Present or absent	Present or absent	Absent	Present	Present	Absent
Claws sheathed by scales	Absent	Present	Present	Present	Present	Present	Present
Lateral scales of claw sheaths	Absent	Long, curved	Long, curved	Short, shell-shaped	Short, shell-shaped	Short, shell-shaped	Short, shell-shaped
No. of precloacal pores in males	0	17–46	16–33	10–11	10	10–13	9–12
Posterior shape of nuchal loop	Rounded	Protracted or rounded	Protracted	Rounded	Rounded	Rounded	Rounded
No. of body bands between nuchal loop and the caudal constriction	3 or 4	3 or 4	3, 4 or 5	2,3 or 4	4	4	4
Dark spotting in body bands	Present or absent	Present or absent	Present or absent	Absent	Present or absent	Absent	Absent
Lateral spotting on belly present or absent	Absent	Absent	Present or absent	Present	Present	Present	Present

#### Distribution and ecology.

Currently, *Goniurosaurus gollum* sp. nov. is known only from Huaiji County, Guangdong Province, China. All individuals were found within a barren limestone cave approximately 50 m from the cave entrance at night after 2130 hrs (Fig. 5). The surface of the cave is covered with bat (unidentified) and bird (*Apus pacificus*) droppings. Stalactites are suspended from the roof and there is no vegetation. *Duttaphrynus melanostictus* was observed in the same area.

## Discussion

With the description of *Goniurosaurus gollum*, there are now 23 known species in the eublepharid genus *Goniurosaurus*, 15 of which occur in China. The *G. yingdeensis* group is endemic in Guangdong Province, with only two species recognized previously, but the discoveries of *G. varius* (Qi et al. 2020) and *G. gollum* adds to this and indicates there might be more new species yet to be discovered.

Although the species of *Goniurosaurus* are also called “cave geckos”, they prefer inhabiting the forest floor, limestone cliffs, rocky and cement drains along the road, and near the entrances of caves. However, the cave-dwelling species *G. gollum* appears to be a true cave dweller as opposed to other *Goniurosaurus*. The fact that all three specimens were found at least 50 m inside the cave from its entrance supports this hypothesis.

These results support the growing body of evidence from China (Luo et al. 2016) and elsewhere (Grismer et al. 2016 and references therein) that karstic habitats not only provide a substrate for the evolution of new species–especially gekkotan lizards–but maintain levels of biodiversity that rival that of other tropical habitats. Yet they are some of the most imperiled and least protected habitats in the world (Day and Urich 2000; Gillieson 2005). The discovery of yet another new species in the vast karstic landscape of northern Guangdong underscores this fact and brings into sharp focus the urgent need to protect these unique landscapes. We aim to protect *G. gollum* from the pet trade, by withholding its precise locality. However, these data are available to qualified researchers upon official request to The Museum of Biology, Sun Yat-sen University.

## Supplementary Material

XML Treatment for Goniurosaurus gollum

## Appendix

**Examined specimens**

*Goniurosaurus bawanglingensis* (*N* = 3): China: Hainan Province: Bawangling National Nature Reserve: SYS r001075, 1670, 2162.

*Goniurosaurus hainanensis* (*N* = 2): China: Hainan Province: Jianfengling National Forest Park: SYS r000349; Baoting County: SYS r001270.

*Goniurosaurus liboensis* (*N* = 3): China: Guizhou Province: Libo County: Maolan National Nature Reserve: SYS r000217, 854, 855.

*Goniurosaurus luii* (*N* = 4): China: Guangxi Zhuang Autonomous Region: Jingxi City: SYS r000255, 256, 859, 860.

*Goniurosaurus varius* (*N* = 5): China: Guangdong Province: Yangshan County: SYS r002330, 2331, 2333, 2362, 2363.

*Goniurosaurus yingdeensis* (*N* = 10): China: Guangdong Province: Yingde City: SYS r000501, 503, 504, 535, 536, 550, 1271, 1272, 1493, 2115.

*Goniurosaurus zhelongi* (*N* = 5): China: Guangdong Province: Yingde City: SYS r000816, 1491, 1492, 2011, 2108.

*Goniurosaurus zhoui* (*N* = 2): China: Hainan Province: SYS r002213, 2214.
